# Characterization of HicAB toxin-antitoxin module of *Sinorhizobium meliloti*

**DOI:** 10.1186/s12866-018-1382-6

**Published:** 2019-01-10

**Authors:** Manon Thomet, Annie Trautwetter, Gwennola Ermel, Carlos Blanco

**Affiliations:** Ribosome, bacteria and stress Team, Univ. Rennes, CNRS, Institut de Génétique et de Développement de Rennes (IGDR), UMR6290, F35000 Rennes, France

**Keywords:** Toxin antitoxin, *hicAB*, BACTH, DNA binding, *Sinorhizobium meliloti*, RNase

## Abstract

**Background:**

Toxin-antitoxin (TA) systems are little genetic units generally composed of two genes encoding antitoxin and toxin. These systems are known to be involved in many functions that can lead to growth arrest and cell death. Among the different types of TA systems, the type II gathers together systems where the antitoxin directly binds and inhibits the toxin. Among these type II TA systems, the HicAB module is widely distributed in free-living Bacteria and Archaea and the toxin HicA functions via RNA binding and cleavage. The genome of the symbiotic *Sinorhizobium meliloti* encodes numerous TA systems and only a few of them are functional. Among the predicted TA systems, there is one homologous to HicAB modules.

**Results:**

In this study, we characterize the HicAB toxin-antitoxin module of *S. meliloti*. The production of the HicA of *S. meliloti* in *Escherichia coli* cells abolishes growth and decreases cell viability. We show that expression of the HicB of *S. meliloti* counteracts HicA toxicity. The results of double hybrid assays and co-purification experiments allow demonstrating the interaction of HicB with the toxin HicA. Purified HicA, but not HicAB complex, is able to degrade ribosomal RNA in vitro. The analysis of separated domains of HicB protein permits us to define the antitoxin activity and the operator-binding domain.

**Conclusions:**

This study points out the first characterization of the HicAB system of the symbiotic *S. meliloti* whereas HicA is a toxin with ribonuclease activity and HicB has two domains: the COOH-terminal one that binds the operator and the NH2-terminal one that inhibits the toxin.

**Electronic supplementary material:**

The online version of this article (10.1186/s12866-018-1382-6) contains supplementary material, which is available to authorized users.

## Background

Toxin antitoxin modules (TA) play important roles in plasmids and prophage stability and are also important actors in bacterial physiology [1]. Canonical TA modules encode a bacterial toxin and a more labile antitoxin in an operon, the antitoxin typically represses the transcription of the operon. The degradation of the antitoxin relieves repression and releases the toxin [2]. The antitoxin could be an RNA molecule (types I and III of TA systems) that controls the level of the toxin protein by either blocking translation of the toxin mRNA or impeding the toxin protein. The nature of the antitoxins may also be proteins that inhibit the toxin by direct interaction or as a hindrance to the effect on the targets (types II and IV of TA systems) [1]. Another types V and VI were described recently. The antitoxin GhoS, which cleaves the mRNA of the toxin GhoT, is classified in the type V of TA system [3]. The type VI corresponds to the antitoxin that presents the toxin to the proteolytic complex ClpXP [4].

The expressions of TA modules are induced by stress [5]. The links between TA modules and adaptation to stresses are numerous such as the coordination of metabolism with the external supply of nutrients [6]. Whereas the TA systems remain quiescent under favourable growth conditions because of the antagonist action of the antitoxin, under stress, the antitoxins are degraded, allowing the toxins to inhibit essential cellular processes. This inhibition ensues in rapid growth arrest [7, 8]. The actions of the toxins are diversified: cleavage, modification and degradation of the cellular targets and, finally hamper the bacterial physiology. Numerous toxins are ribosome-dependent or -independent endonucleases. Other actions of the toxins are described such as the post-translational modification of the targets or the depolarization of the bacterial membrane leading to the arrest of the ATP synthesis [1]. The toxins could have either indirect effects i.e. MqsR, which is a ribosome independent mRNA interferase, cleaves the *ygiS* mRNA resulting in increased bile tolerance [9]; or domino effects where one toxin could control another TA module: MqsR cleaves antitoxin *ghoS*-*ghoT* mRNA in the *ghoS* coding region leading to the enhancement of *ghoT* coding region [10]. The physiological consequence of TA expression could also result of a direct role of the antitoxin on targets other than its cognate toxin: MqsA and DinJ antitoxins are able to repress *rpoS* gene, affecting the general stress response [11, 12]. TA modules are involved in the virulence of *Salmonella* [13], *Haemophilus influenza* [14] and *Staphylococcus aureus* [15]. Many of them are implicated in persistence [1] and are considered as targets for antibacterial development. However, the evidence for a role of TA systems in persister cell formation is controversial [16–18].

The HicAB system belonging to the type II TA systems are found in many bacteria and archaea and has been shown to be involved in the stress response virulence and persistence [19–22]. The first identification of *hicAB* was done in *Haemophilus influenza* [23] and the term *hic* came from the localization near the pathogenicity island *hif*. Then *hicAB* operons were identified in other bacteria such as *Escherichia coli*, *Burkholderia pseudomallei*, *Yersinia pestis*, *Pseudomonas aeruginosa* and the structure of HicAB was determined in *Yersinia pestis* and *Streptococcus pneumoniae* [19, 24–26]. In *E. coli* K12, the *hicAB* locus encodes the toxin HicA that cleaves mRNAs and also the tmRNA by a ribosome-independent manner, and the antitoxin HicB [27].

In *Sinorhizobium meliloti* 53 TA modules were predicted [28], only a few of them were characterized. NtpP/R and VapB/C are involved in symbiotic efficiency [29, 30]. Systematic deletion of TA loci in megaplasmids pSymA and pSymB shows that the deletion of some of them affects growth [31], suggesting that part of them is not functional. In this study, we focused on the TA module HicAB of *S. meliloti* corresponding to SMc04441 and SMc04269 hypothetical proteins as defined by Capela et al., [32]. The HicAB modules are highly subjected to horizontal gene transfer and are widely distributed in free-living Bacteria- and Archaea-species, and are not found in genomes of obligate parasites and symbionts [26] such as *S. meliloti*. Moreover, this HicAB module is involved in virulence and adaptative traits: attenuated virulence of *Y. pestis* mutants [33] development of persister cells in the bacteria *B. pseudomaellei* [19]; adaptation to extracellular stresses in *E. coli* [21]. In this study, we show that the operon consisting of the ORFs SMc04441 and SMc04269 in *S. meliloti* encodes a TA module composed of the functional toxin HicA and antitoxin HicB.

## Results

### *hicAB* of *S. meliloti* encodes a functional TA system

In order to study the HicAB module of *S. meliloti*, we assumed that several TA systems could be active in heterologous bacteria [34]. Considering this hypothesis that the TA systems possess a relatively broad host range, we examined the functionality of the HicAB module of *S. meliloti* by constructing a conditional system for the expressions of HicA and HicB in *Escherichia coli*.

**Fig. 1:**
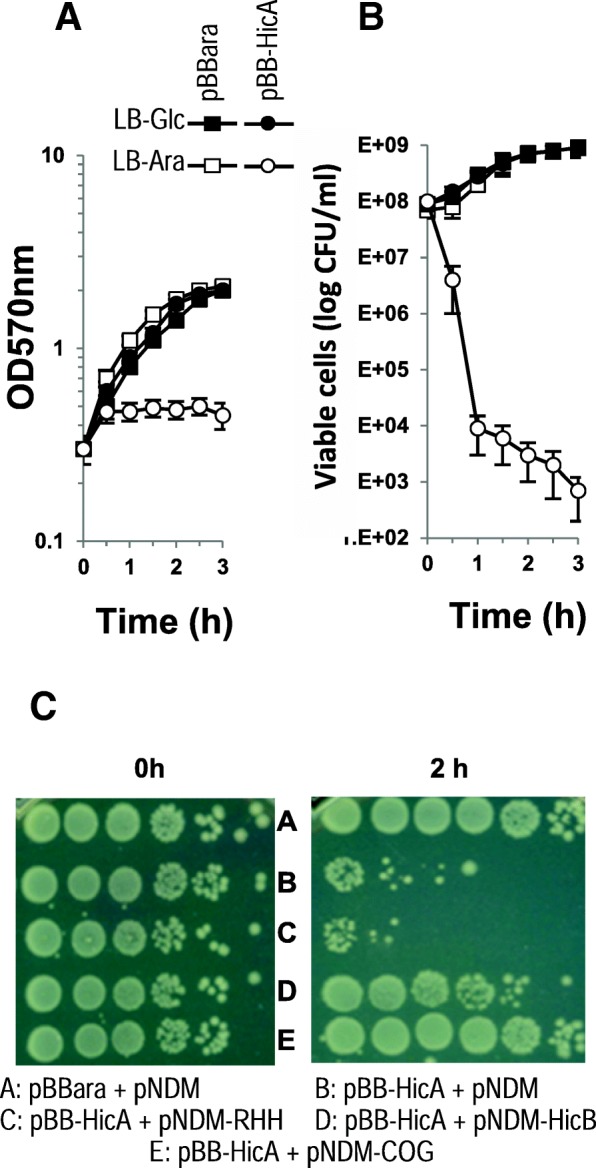
HicB prevents the toxicity of HicA in *E. coli*. *E. coli* strain MG1655 containing pBBara (squares) or pBB-HicA (circles) were precultured in LB medium containing 10 mM glucose to an OD_570nm_ of 0.2. After centrifugation, cells were suspended in LB medium containing 10 mM glucose (black symbols) or 10 mM arabinose (white symbols). The growth of the strains was then followed for 3 h and estimated using either turbidity (OD_570nm_, **a**) or viable cell counts (log (CFU/mL), **b**). The panel **c** corresponds to the numeration of the *E. coli* MG1655 cells containing pBBara + pNDM (A), pBB-HicA + pNDM (B), pBB-HicA + pNDM-RHH (C), pBB-HicA + pNDM-HicB (D), pBB-HicA + pNDM-COG (E) after 2 h of arabinose induction in LB medium, the details of the procedure were as in A. Tenfold dilutions were spotted on a LB plate and incubated for 48 h

The *hicA* ORF of *S. meliloti*, cloned in the pBB-HicA allows the production of HicA under control of the P_BAD_ promoter. After induction with arabinose, the influence of the expression of HicA on cell growth was investigated in *E. coli* strain MG1655 (Fig. 1). Cells carrying the empty pBBara expression vector showed no difference in growth or in viability when they were either induced or not by arabinose. When cells harbouring pBB-HicA were grown without arabinose, they exhibited the same growth pattern estimated by turbidity (OD_570nm_) but when HicA production was induced, growth was abolished (Fig. 1a). Moreover, the number of CFU dramatically reduced after induction and continuously decreased throughout time representative of the lethal effect of the toxin (Fig. 1b).

To investigate whether HicB could alleviate HicA toxicity, *hicB* ORF was cloned in the plasmid pNDM220. This vector is compatible with pBBara and the expression of HicB is inducible with IPTG. Plasmid derivatives from both vectors were introduced in *trans* and induced simultaneously in *E. coli*. After two hours of induction, cells carrying the empty vector pNDM220 and pBB-HicA exhibited lethality (Fig. 1c, line B) whereas in cells harbouring pBB-HicA and pNDM-HicB for which the simultaneous production of HicA and HicB reduced drastically HicA lethal effect (Fig. 1c, line D).

### HicB binds to *hicAB* promoter region

**Fig. 2:**
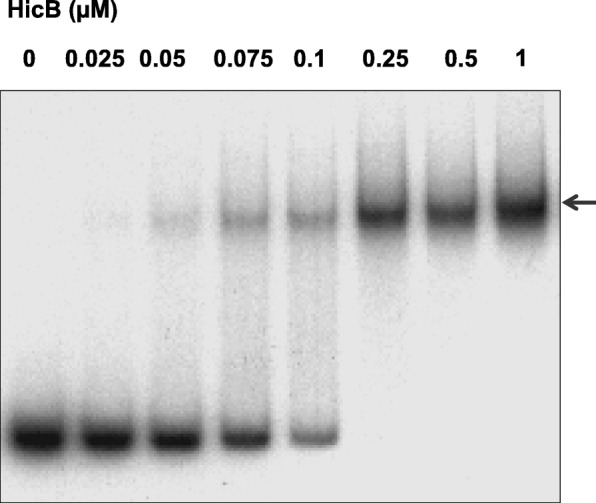
HicB binds hicAB promoter region. Gel mobility shift analysis of specific binding of the purified HicB to the promoter region of *hicAB* operon. The probe, a 223 bp DNA fragment amplified by PCR, was incubated with increasing amounts of purified HicB (0 to 1 μM) and submitted to electrophoresis in a 5% PAGE in Tris acetate buffer pH 7.5. The position of the protein-DNA complex is indicated by an arrow

In type II TA systems the transcription of both toxin and antitoxin are autoregulated: the antitoxin binds to the promoter of the TA operon and thus inhibits transcription of both toxin and antitoxin [1]. To study HicB interaction with *hicAB* promoter, we performed an electrophoretic mobility shift assay (EMSA) using purified His-tagged HicB and a 223 bp sequence containing the promoter of genes *hicAB*, which was previously characterized [35, 36] (Fig. 2). An upshifted band appeared faintly with a low concentration (50 nM) of HicB and was clearly present with the highest concentrations (from 0,25 to 1 μM) of HicB. These results demonstrate that the HicB protein of *S. meliloti* binds to the *hicAB* promoter region.

### HicB interacts with HicA

**Fig. 3.**
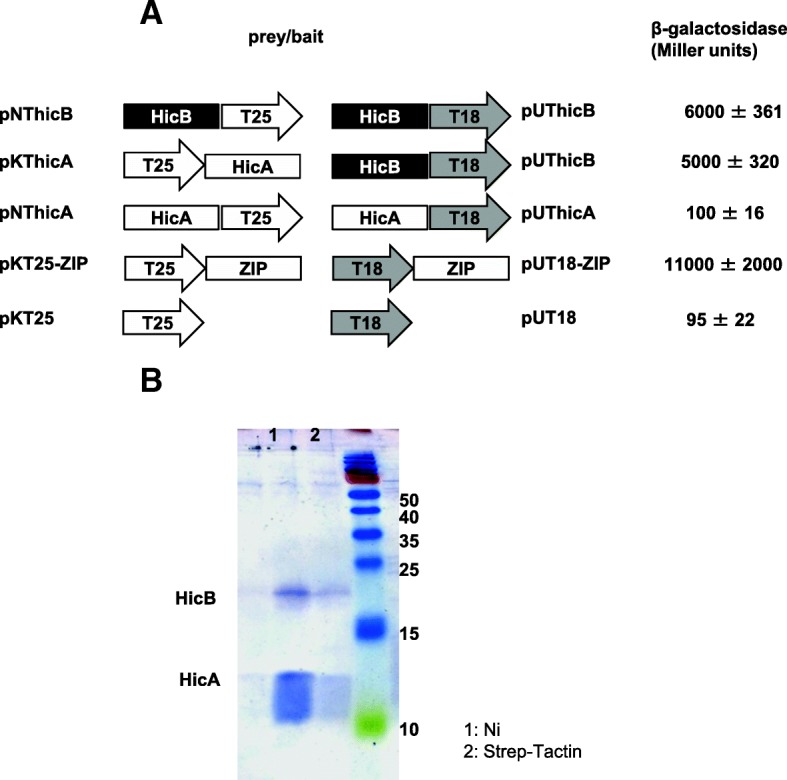
HicB interacts with HicA. **a** Two hybrid analysis of HicA and HicB interaction. HicB (black squares) and HicA (white squares) were fused to T25 (white arrow) or T18 (grey arrow) catalytic domains of *Bordetella pertussis* adenylate cyclase both at the N or C terminus of these domains. The whole set of combinations is presented in Fig.S1. For this purpose *hicA* and *hicB* open reading frames were amplified by PCR using ToxXba-ToxEco and DopXba-DopEco primers (Table 3). The amplicons cleaved by *Xba*I and *Eco*RI were cloned into pKT25, pKNT25, pUT18 and pUT18C, the resulting plasmids are listed in Table 2. After introduction of the different recombinant plasmids into *E. coli* strain BTH101, β-galactosidase activities were assayed after growth in LB medium containing 1 mM IPTG. Empty vectors pUT18 and pKNT25 were used to determine basal level of β-galactosidase activity and pKT25-Zip and pUT18-Zip as positive control of a high interaction. β-galactosidase activity (Miller units) is indicated, results are the average of three independent experiments. **b**. SDS-PAGE of both HicA-Strep and HicB-6His. A synthetic operon *hicA*-strep, *hicB*-6His was cloned in pET22b(+). *E. coli* BL21 containing this plasmid was grown in LB medium to an OD_570nm_ of 0.6, 1 mM IPTG was added, and the growth of cells was carried on three hours after induction. Cells were collected and broken, protein extract was applied to Ni affinity chromatography using imidazole for elution (lane 1) and Strep-Tactin affinity chromatography using desthiobiotin for elution (lane 2). The eluted proteins were resolved on a 12.5% SDS PAGE.

To assess HicA-HicB protein interactions, we used the bacterial two-hybrid system (BACTH) in *E. coli cya* strain BTH101. BACTH relies on the recovery of *Bordetella pertussis* adenylate cyclase whose domains T18 and T25 are split and can be fused with proteins [37]. HicA and HicB were alternatively fused to the NH2 and COOH ends of T18 and T25 domains in different combinations, for which β-galactosidase assays were monitored (Additional file 1: Figure S1). All the combinations between HicA and HicB showed significant β-galactosidase activities in regards to those determined with empty vectors without any fused protein and self-interactions of HicA. High β-galactosidase activity was detected in cells producing T25-HicA and T18-HicB reflecting HicA-HicB interaction (Fig. 3a). Although HicA does not self-interact the co-expression of T25-HicB and T18-HicB resulted in high levels of β-galactosidase activity (Fig. 3a) suggesting HicB self-interaction and a possible dimeric organization of the protein.

To confirm HicA-HicB interaction, we performed co-purification experiments. A synthetic *hicAstrep-hicBhis* operon (Additional file 2: Figure S2) was introduced in pET22b(+) vector leading to the productions of strep-tagged HicA and his-tagged HicB at their COOH ends. After IPTG induction, the produced proteins were alternatively purified on IMAC chromatography and on Strep-Tactin resins. In both cases HicA and HicB were co-purified (Fig. 3b).

These data indicate that *S. meliloti* HicA and HicB interact, as expected for a toxin-antitoxin system. Moreover HicB would be a multimeric protein while HicA behaves as a monomer as this has been shown using the two-hybrid assays and described for HicAB in *Y. pestis* [24].

### Antitoxin stability and toxin purification

**Fig. 4:**
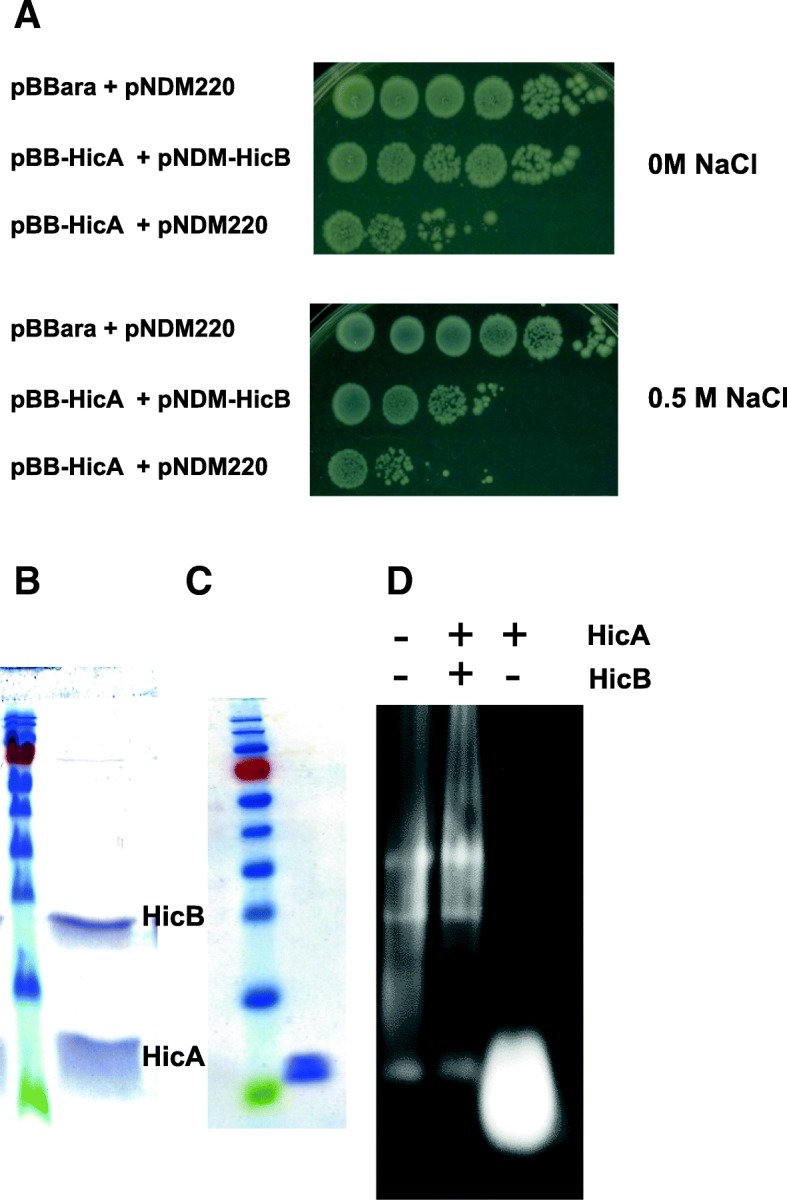
HicB stability and RNase assay. Viable cell counts of cells of *E. coli* strain MG1655 containing pBBara and pNDM220, pBB-hicA and pNDM-hicB or pBB-hicA and pNDM220 that were grown in M63 medium containing or not 0.5 M NaCl (**a**). Strains were induced with 1 mM arabinose and 1 mM IPTG. Viable cell counts (ten fold dilutions) were determined throughout time, the figure illustrates results obtained 3 h after induction. Cells of *E. coli* strain BL21 containing pETHicAStrep-HicBHis grown in M63 medium were induced with 1 mM IPTG for 3 h. Half of the culture was centrifuged and protein content was loaded on Strep Tactin and eluted with 1 mM desthiobiotin (**b**). The other half of the culture was added with 0.5 M NaCl and incubation was extended for 4 h, then the protein content was loaded on Strep-Tactin and eluted with desthiobiotin (**c**). 20 μg of eluted proteins were solved by SDS PAGE (**b, c**). The same amount of eluted proteins was incubated with ribosomal RNAs 1 h at 37 °C and then loaded on 1% bleach agarose gel (**d**).

Toxins are stable proteins while antitoxins are targeted by proteases such as the Lon protease or by ClpP associated with either ClpA or ClpX [2]. Proteases synthesis is induced by stresses such as osmotic and starvation ones. Thus we hypothesized that the strains expressing toxin and antitoxin must have their growth affected by stress while it would not be affected in non-stressing medium. The *E. coli* strain MG1655 containing both pBB-HicA and pNDM-HicB, or both pBB-HicA and pNDM220, or both pBBara and pNDM220 vectors as controls, was grown in M63 medium after induction of *hicA* and *hicB*. The sole induction of HicA and HicB production did not affect growth (data not shown). A growth arrest was observed when osmotic stress (addition of NaCl to media) was applied to strains with induced expressions of toxin and antitoxin (Fig. 4 a). The strains containing empty vectors still grew (data not shown). These results showed that the cell survival was affected after stress application. Concerning the strain containing both pBB-HicA and pNDM220, defect of growth was observed after arabinose induction leading to the production of the toxin HicA. A concentration of 0.5 M NaCl in M63 medium amplified this defect of growth.

The extraction of proteins was performed in *E. coli* BL21 cells harbouring pETHicAStrepHicBHis 3 h after IPTG induction, and 4 h after NaCl addition (i.e. 7 h after induction); proteins were loaded on Strep-Tactin column that traps the strep-tagged toxin. Toxin and antitoxin were eluted from extracts of non-salted cultures (Fig. 4 b) while only toxin was eluted from salted cultures (Fig. 4 c). These data show that the antitoxin produced before NaCl addition is degraded during salt stress adaptation.

To determine the toxic effect of HicA, RNase activity of purified proteins was analysed. Extracts of cells cultured in M63 medium that contained the HicA-HicB complex did not show RNase activity against purified ribosomal RNAs. In contrast ribosomal RNAs were fully degraded by purified HicA originated from cells cultured in M63 medium containing 0.5 M NaCl after 1 h at 37 °C (Fig. 4 d).

These results show that contrary to HicB, HicA ribonuclease is not degraded by proteases induced by osmotic stress [1, 2]. This allows a simple toxin purification protocol that did not need protein denaturation and renaturation as described for other HicA proteins.

### HicB is composed of two functional modules

The structure of HicAB was determined in *Y. pestis* and *S. pneumoniae*. In both bacteria, HicA and HicB structures are very close [24, 25]. In these bacteria, HicA is a monomer while HicB is dimeric or tetrameric. This antitoxin is composed of two domains linked by a hinge, a NH2 domain interacting with HicA toxin and a COOH domain possessing a ribbon-helix-helix (RHH) motif that interacts with the operator. HicB3 of *Y. pestis* dimerizes through the RHH domain while HicB of *S. pneumoniae* dimerizes through the NH2 domain.

Clustal alignments of *S. meliloti* HicA with HicA of *E. coli*, *Y. pestis* and *S. pneumoniae*, show that all the proteins are very similar (Additional file 3: Figure S3), the histidine conserved residue important for the activity is conserved in *S. meliloti* HicA. In contrast, Clustal alignments of HicB show that the amino acids are less conserved in HicB proteins (Fig. S3), the DNA binding domain is not conserved, and only 6 aminoacids are identical in the four proteins, nevertheless no particular function was attributed to them in structural studies [24, 25]. Structure prediction of *S. meliloti* with Phyre server shows that its structure is close to that of *Y. pestis* and *S. pneumoniae*, main differences were observed in the hinge region (Additional file 4: Figure S4 and Additional file 5: Figure S5).

**Fig. 5:**
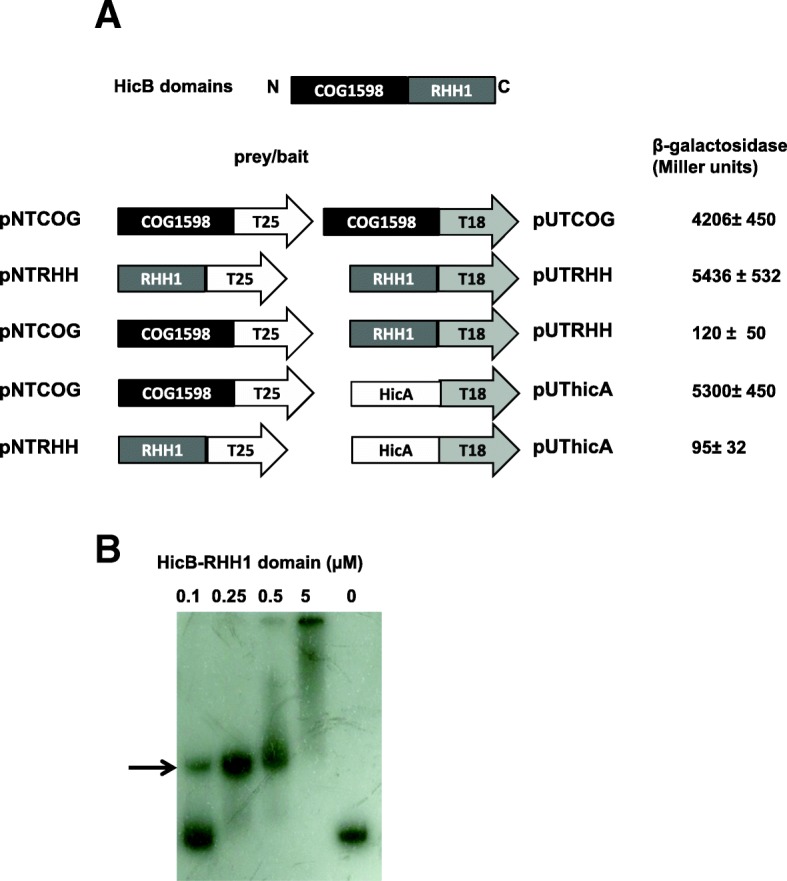
Functionalities of HicB domains. **a** DNA fragment coding for NH2 domain (COG1598) and COOH domain (RHH1) of HicB were amplified by PCR and introduced into pUT18, pUT18C, pKT25 and pKNT25 vectors allowing fusion of these domains with T25 and T18 fragments of *B. pertussis* adenylate cyclase (All the combinations are shown in Fig. S6). Plasmids were introduced in *E. coli* strain BTH101, and the cells were grown in LB medium containing 1 mM IPTG and β-galactosidase activity was assayed. **b** RHH1 domain binds operator sequence. Increasing amounts (0 to 5 μM) of RHH1 domain fused to 6 His were incubated with the promoter region of *hicAB* operon (223 bp PCR fragment used in Fig. 2). Then a 5% PAGE in Tris acetate buffer pH 7.5 was performed to resolve the protein DNA complexes (indicated by an arrow).

The two domains (RHH for C-terminal and COG for N-terminal domains) were cloned in pBBara, the hinge region was conserved in both constructs. The arabinose induction of the two domains does not induce toxicity in *E. coli* cells (data not shown). Thus, the sequences corresponding to the two domains were separately cloned into pNDM vector and introduced in *E. coli* with pBB-HicA. After induction with arabinose and IPTG, toxicity was observed when HicA and HicB-RHH were produced simultaneously (Fig. 1 c lane C). In contrast expression of HicB-COG domain abolishes HicA toxicity (Fig. 1c lane E). The antitoxin effect is greater than that observed with HicB (Fig. 1c lane E). Interaction of the HicB NH2-domain with HicA was confirmed by BACTH analysis (Fig. 5 and Additional file 6: Figure S6). The HicB COG domain must multimerize as deduced from the high ß-galactosidase activity observed in HicB-COG self-interaction in BACTH assays (Fig. 5 and Additional file 5: Figure S5). Moreover, this HicB-COG was unable to interact with HicB-RHH domain in BACTH assays. The HicB RHH domain was purified and used in EMSA studies with HicB binding region. It bound operator sequence as efficiently as HicB protein (Fig. 5). The analysis of BACTH assays showed that RHH domain dimerizes, a feature necessary for efficient binding to the operator.

## Discussion

Numerous TA modules exist in *Bacteria*, they are related to numerous functions that allow the control of growth arrest when cells adapt to variable environments. In the nitrogen-fixing soil bacterium *S. meliloti*, numerous TA modules were predicted [26, 28] and one TA module, encoded by *smc04441*/*smc04269*, showed homologies with *hicAB* loci [32]. In this study, we demonstrate that the HicAB module of *S. meliloti* is functional in *E. coli*. The expression of HicA toxin under the control of the arabinose promoter in *E. coli* permits us to show that HicA toxin provoked a decrease of the viability of bacteria prevented by the simultaneous expression of HicB antitoxin (Fig. 1c). The interactions between the two proteins were confirmed by the two hybrid assays using the BACTH system [37] whatever the combination using HicA or HicB (Fig. 3a and Additional file 1: Figure S1). The co-purification of strep-tagged HicA and His-tagged HicB using affinity chromatographies on either Nickel or Strep-Tactin resins is another argument in favour of HicA-HicB interaction (Fig. 3b). Furthermore, these experiments allow us to reveal a self-interaction and a possible dimerization of HicB (Additional file 1: Figure S1). These data indicate that *S. meliloti* HicA and HicB interact, as expected for a toxin-antitoxin system. Moreover there are evidence that HicB would be a multimeric protein while HicA behaves as a monomer, as described for HicAB in *Y. pestis* [24]. The purified His-tagged HicB bound to the promoter of genes *hicAB* (Fig. 2a). Thus the HicB protein of *S. meliloti* binds to the *hicAB* promoter region such as the antitoxins HicB of other bacteria [24, 38].

The simultaneous induction of HicA and HicB affected growth only when osmotic stress was applied by the addition of NaCl to the medium (Fig. 4a), meaning that the HicB antitoxin was digested by osmotic stress-induced proteases such as Lon or ClpP as this phenomenon has been observed for other antitoxins [1, 2]. This particularity was used to purify the HicA toxin only (Fig. 4c) using a simple protocol. The HicA protein of *S. meliloti* was shown to be a ribonuclease that degraded purified ribosomal RNAs (Fig. 4d). RNA cleavage would block translation and cause primarily a growth arrest and finally a decrease of cell viability [27].

The analysis of the predicted structure of HicB showed a NH2 domain with homologies to the domain of HicB of *S. pneumoniae* and *Y. pestis*, interacting with HicA toxin and a COOH domain possessing a potential ribbon-helix-helix motif, binding promoter that is less conserved (Additional file 2: Figure S2). The different experiments permitted to show that the two domains are active independently: the NH2 domain of HicB interacts with the toxin HicA and the COOH domain of HicB binds to the promoter of *hicAB* operon. Moreover, these two domains potentially showed self-interaction and a possible dimerization. HicB of *Y. pestis* and *S pneumoniae* possess only one dimerization domain, but not in the same region. Isolated domains of *S. meliloti* have both the hinge region, nevertheless we could exclude that it is responsible for dimerization since no interaction was observed between NH2 and COOH domains. Thus *S. meliloti* HicB must have a slightly different structure with two interaction regions between monomers. Alternatively, HicB of the other bacteria also has the same ability to dimerize through two regions, but only one is used depending of experimental conditions that were different for *Y. pestis* and *S. pneumoniae* [24, 25].

## Conclusion

In this study we characterize the biochemical properties of HicA and HicB proteins of *S. meliloti*. Purified HicA has an RNAse activity; HicB is a DNA binding protein. Both proteins interact and this interaction via the NH2 domain of HicB abolishes the RNAse activity of the toxin. The following work, which is in progress, would have to determine the physiological importance of HicAB TA module in *S. meliloti*.

## Methods

### Bacterial strains and culture conditions

*E. coli* and *S. meliloti* strains are listed in Table 1. They were grown in LB medium at 37 °C. For the selection of *E. coli* transformants, antibiotics (Sigma-Aldrich) were added: ampicillin (50 μg/mL), kanamycin (50 μg/mL), neomycin (50 μg/mL), chloramphenicol (25 μg/mL) and tetracycline (10 μg/mL). For the culture of *S. meliloti* strain 102F34, streptomycin was used at 100 μg/ml.

**Table 1:** Bacterial strains

Strains	Genotypes	References
*S. meliloti*		
102F34	WT SmR	[45]
*E. coli*		
MG1655	F^−^, lambda^−^, rph-1	[46]
BL21	*E. coli* B F^−^ *dcm ompT hsdS gal*λ(DE3)	[47]
BTH101	F′*, cya-99, araD139, galE15, galK16, rpsL1 (Str*^*R*^*), hsdR2, mcrA1, mcrB1, relA1*	[48]

### DNA manipulations

Chromosomal and plasmid DNA isolations were undertaken according to the standard procedures [39]. Sequencing was performed by Eurofins.

### Plasmids, synthetic genes and primers

The plasmids and oligonucleotides that were used in this study are listed in Tables 2 and 3. The synthetic genes are described in Additional file 2: Figure S2.

**Table 2:** Plasmids used in this study

Names	Genotypes	References
pBAD24	ori ColE1 *araC*, P_BAD_ *bla*	[49]
pNDM220	Mini R1, *bla, lacI*^*q*^, P_A1/O4/O3_	[50]
pBBR1-MCS2	ori pBBR1, *lacZα, aphA*	[40]
pET22b(+)	ori ColE1, *bla, lac*I, T7p	Novagen
pKT25	ori P15A, *aac*, T25	[37]
pKNT25	ori P15A, *aac*, T25	[37]
pUT18	ori ColE1 *bla*, T18	[37]
pUT18C	ori ColE1 *bla*, T18	[37]
pKT25-Zip	ori P15A, *aac*, T25:: Zip	[37]
pUT18-Zip	ori ColE1 *bla*, T18:: Zip	[37]
pBBara	pBBR1-MCS2, *araC*, P_BAD_	
pBB-HicA	pBBR1-MCS2, *araC*, P_BAD_ -*hicA*	This study
pET-HicB	pET22 *hicB* 6 his	This study
pET-RHH	pET22 RHH1 6 his	This study
pET-COG	pET22 COG 6 his	This study
pETHicAStrepHicBHis	pET22 synthetic operon	This study
pNDM-HicB	pNDM220 *hicB*	This study
pNDM-COG	pNDM220 NH2 fragment of HicB (COG1598)	This study
pNDM-RHH	pNDM220 COOH fragment of HicB (RHH)	This study
pKThicA	pKT25 *hicA*	This study
pKThicB	pKT25 *hicB*	This study
pNThicA	pKNT25 *hicA*	This study
pNThicB	pKNT25 *hicB*	This study
pUThicA	pUT18 *hicA*	This study
pUThicB	pUT18 *hicB*	This study
pUChicA	pUT18C *hicA*	This study
pUChicB	pUT18C *hicB*	This study
pKTCOG	pKT25 COG1598	This study
pNTCOG	pKNT25 COG1598	This study
pKTRHH	pKT25 RHH1	This study
pNTRHH	pKNT25 RHH1	This study
pUTCOG	pUT18 COG1598	This study
pUCCOG	pUT18C COG1598	This study
pUTRHH	pUT18 RHH1	This study
pUCRHH	pUT18C RHH1	This study

**Table 3:** Oligonucleotides used in this study

Names	Sequences*	Restriction sites
Tox pBAD-A	TGGGCTAGCGTGTGTATTGTCGTATCAGATG	*Nhe*I
Tox pBAD-B	TGCCGTCGACTTACCTCAATTTCAAACC	*Sal*I
HicBNde	GAGGTAAGCATATGCGCAAC	*Nde*I
HicBXho	GGTATCTCGAGCACATTTTTG	*Xho*I
RHHNde	AGGTTCATATGTCCGACGCC	*Nde*I
ToxXbaT	CATCTAGAGAGCGGCGAC	*Xba*I
ToxEcoT	GAATTCCTCAATTTCAAACCG	*Eco*RI
DopXbaT	CCTCTAGACAACTATATCGG	*Xba*I
DopEcoT	AGGAATTCACATTTTTGCTGG	*Eco*RI
RHHXba	AATCTAGAATGTCCGACGCCGAGAACAGG	*Xba*I
COGEco	TAGAATTCCAACCTCAAGGGAGGAGGG	*Eco*RI
RHHNde	CGGTGAAAACATATGCAAAAAGAG	*Nde*I
HicBNH2Nde	GGCATATGCGCAACTATATCGGATTGATC	*Nde*I
HicBNH2Xho	CCCTCGAGTTCGGCGAAGGCATCTATC	*Xho*I
PET-Bgl	GGGGGGGAAGATCTAGAAATAATTTTGTTTAAC	*Bgl*II
PET-EcoRI	CGGGAATTCAGCAAAAAACCCCTCAAG	*Eco*RI
EMSAD	GATCCGACGGTTCGAGACCATCC	none
EMSAR	TCTTCGGGTGAGGAACGGTAACC	none

*the restriction sites are underlined

A new vector, pBBara was constructed: the *Nsi*I-*Hin*dIII fragment carrying *araC*, *araO* was liberated from pBAD24 and introduced between the corresponding sites of pBBR1-MCS2, which was reported to have five to ten copies per chromosome/cell in *E. coli* [40].

In order to express HicA, the *hicA* sequence was amplified from genomic DNA of *S meliloti* strain 102F34 using the primers toxpBAD-A (carrying a *Nhe*I site) and toxpBAD-B (carrying a *Sal*I site). After restriction, the PCR fragment was introduced between the corresponding sites of pBBara, giving the pBB-HicA plasmid.

For the expression of HicA-StrepTag and HicB-6HisTag, a synthetic *hicAB* operon was synthesized (Eurofins Genomics) (Additional file 2: Fig. S2) with a *Nde*I restriction site at the initiation codon of *hicA*, a Strep-Tag (WSHPQFE) at the COOH extremity of *hicA*, a *Xho*I site at stop codon of *hicB* in order to allow the expression of a 6His-Tag after the insertion of the fragment into the pET22b(+) vector between the *Nde*I and *Xho*I sites. This construct was named pETHicAStrepHicBHis.

The plasmid pET-HicB contains the HicB ORF, which was amplified by PCR from *S. meliloti* genomic DNA using the primers (HicBNde, HicBXho) and inserted into the pET22b(+) vector between the restriction sites *Nde*I and *Xho*I, introducing a 6His-Tag at the COOH extremity of HicB.

The COG1598 (NH2-terminal extremity) and the RHH1 (COOH-terminal extremity) domains of HicB were obtained using amplification with HicBNH2Nde/HicBNH2Xho and RHHNde/HicBXho respectively and introduced into pET22b(+) vector yielding pET-COG and pET-RHH respectively.

Primers PET-BglII and PET-EcoRI were used to amplify the sequence of interest from pET-HicB, pET-RHH and pET-COG1598. The amplicons were digested with *Bgl*II and *Eco*RI and cloned between the *Eco*RI and *Bam*HI sites of pNDM220, yielding pNDM-HicB, pNDM-COG and pNDM-RHH respectively.

### Purification of HicA and HicB

The pET22b(+) expression vector possesses a T7 promoter controlled by LacI. All its derivatives were transformed in BL21 cells. pET-HicB, pET-RHH and pET-COG1598 transformants were grown in LB medium to an OD_570nm_ of 0.6, IPTG was added at 1 mM and incubation was continued for 4 h. Cells were collected by centrifugation (5000 g, 15 min), washed (Tris-HCl 20 mM, NaCl 500 mM, imidazole 5 mM, pH 8), resuspended in the same buffer, broken in a French press (8,6 10^6^ N m^− 2^) and centrifuged (20,000 g, 30 min). Protein extracts were loaded on a Ni-NTA agarose resin (Qiagen), washed with 60 mM imidazole buffer and proteins eluted with 200 mM imidazole buffer. Proteins were dialysed against PBS buffer.

BL21 containing pETHicAStrepHicBHis was grown in LB medium to an OD_570nm_ of 0.6 before 1 mM IPTG was added for protein induction. For purification of HicA-HicB complex, the bacterial cells were harvested 3 h later. For HicA purification 1 M NaCl was added 3 h after induction with IPTG and incubation was continued for 4 h. The purification steps were identical to those described above. Alternatively protein extract was loaded on a Strep-Tactin resin (IBA Lifesciences), and eluted with 2.5 mM desthiobiothin.

### RNase assay

*E. coli* ribosomes were purified on 10–40% sucrose gradients. Collected fractions containing 70S ribosomes were centrifuged (150,000 g, 2 h) and 70S particles re-suspended in 50 mM TRIS pH 7.5, NaCl 0.75 M, MgSO4 10 mM. RNA was extracted with Trizol (Thermo Fisher Scientific) and re-suspended in PBS buffer.

HicA and HicAB complexes were purified on Strep-Tactin resin (IBA Lifesciences) and resuspended in PBS buffer. Ribosomal RNA (130 pM) and proteins (20 nM) were incubated at 37 °C for 1 h and RNA was analysed on bleach agarose gels [41].

### EMSA

A 221 bp DNA fragment embedding *hicAB* promoter (− 213. + 8) was amplified and labelled using PCR with EMSAD and EMSAR primers in the presence of [α^32]^ P dCTP. The binding reaction was performed in a 20 μl reaction volume containing10 mM TRIS-HCl pH 7.5, 50 mM NaCl, 1 mM DTT, 300 μg ml^− 1^ BSA the labelled DNA fragment and the purified HicB-His6 or RHH-6His.

The reaction was incubated 30 min at 25 °C followed by the addition of 2 μl of 50% glycerol and electrophoresis through a 5% native polyacrylamide gel at 170 V cm^− 1^ in TAE buffer (20 mM Tris, 40 mM acetate, 1 mM EDTA, pH 7.5). The gel was then dried and revealed by autoradiography.

### BACTH analysis

HicA and HicB ORFs were amplified by PCR using ToxXbaT/ToxEcoT and DopXba/DopEcoT primer pairs for HicA and HicB respectively and cloned into pKT25, pKNT25, pUT18 and pUT18C vectors between *Xba*I and *Eco*RI restriction sites. Strain BTH101 was transformed with a combination of one pUT (18 or 18C) derivative and a pKT (or pKNT) derivative in order to obtain all the combinations to analyse HicA-HicA, HicB-HicB and HicA-HicB interactions. pKT25-ZIP and pUT18-ZIP were used as reference for positive interaction and pKT25 and pUT18 as control for the absence of interaction. BTH101 transformants were grown in LB or M63 medium containing 10 mM glycerol as growth substrate. β-galactosidase enzymatic activity was assayed after induction with 1 mM IPTG according to [42].

HicB COG1598 and RHH1 domains were cloned into pKT25, pKNT25, pUT18 and pUT18C using DopXbaT/COGEco and RHHXba/DopEcoT primer pairs. BACTH analysis was performed as described for HicA-HicB interaction.

## Additional files

Additional file 1: **Figure S1.** HicA/HicB interaction analysis using the BACTH system. For analysis of interactions between HicA and HicB, either one of the plasmids pKThicA, pNThicA, pKThicB, or pNThicB were transformed into the *E. coli* strain BTH101 reporter strains (a *cya*-deficient strain), followed by secondary transformation of any of the following: pUThicA, pUChicA, pUThiB, or pUThicB. Positive and negative controls were performed using pKT25-ZIP/pUT18-ZIP and pKNT25/pUT18C sets respectively. Positive interactions allow the reconstitution of adenylate cyclase activity and thus the expression of *lacZ* gene in the *cya* strain BTH101. β–galactosidase activity (Miller units) is the mean of three independent experiments. (PPTX 95 kb)

Additional file 2: **Figure S2.** Nucleotide sequence of *hicA-hicB* synthetic operon introduced into pEt22b(+). HicA residues are highlighted in yellow, strep tag in pink, HicB in green and his tag in red. (PPTX 99 kb)

Additional file 3: **Figure S3.** Alignments of HicA and HicB proteins with HicA and HicB homologues of *E. coli*, *Y. pestis* and *S. pneumoniae* using Clustal Omega [43]. Symbols indicate a conserved residue (*), conservative mutation (:) and a semi-conservative mutation (.). The H23 residue critical for HicA activity [25] is shown in red. (PPTX 55 kb)

Additional file 4: **Figure S4.** Alignments of *S. meliloti* HicB with HicB of *S. pneumoniae* (A)and HicB3 of *Y. pestis* (B) using Phyre [44]. (PPTX 598 kb)

Additional file 5: **Figure S5.** *S. meliloti* HicB structure predicted by Phyre using HicB of *Y. pestis* and *S. pneumoniae* as template. (PPTX 80 kb)

Additional file 6: **Figure S6.** HicB domains interactions. For analysis of auto-interactions between HicB domains and their interaction with HicA, either one of the plasmids pKTCOG, pNTCOG, pKTRHH, pNTRHH, pKThicA or pNThicA were transformed into the *E. coli* strain BTH101 reporter strains (a *cya*-deficient strain), followed by secondary transformation of any of the following: pUTCOG, pUCCOG, pUTRHH, pUTCRHH or pUThicA. Positive and negative controls were performed using pKT25-ZIP/pUT18-ZIP and pKNT25/pUT18 sets respectively (not shown). Positive interactions allow the reconstitution of adenylate cyclase activity and thus the expression of *lacZ* gene in the *cya* strain BTH101. β–galactosidase activity (Miller units) is the mean of three independent experiments. (PPTX 81 kb)

## Abbreviations

BACTH: Bacterial adenylate cyclase two-hybrid
BSA: Bovine serum albumin
CFU: Colony forming units
COG: Clusters of orthologous groups
EMSA: Electrophoretic mobility shift assay
IPTG: Isopropyl β-D-1-thiogalactopyranoside
OD570nm: Optical density measured at 570 nm
ON: Overnight
PBS: Phosphate-buffered saline
PCR: Polymerase chain reaction
RHH: Ribbon-helix-helix
