# Supplementary figures and images for: Characterization of HicAB toxin-antitoxin module of Sinorhizobium meliloti
Source: BMC Microbiol. 2019 Jan 10;19:10. doi: 10.1186/s12866-018-1382-6 (PMC6327479; doi:10.1186/s12866-018-1382-6)

## Slide 1
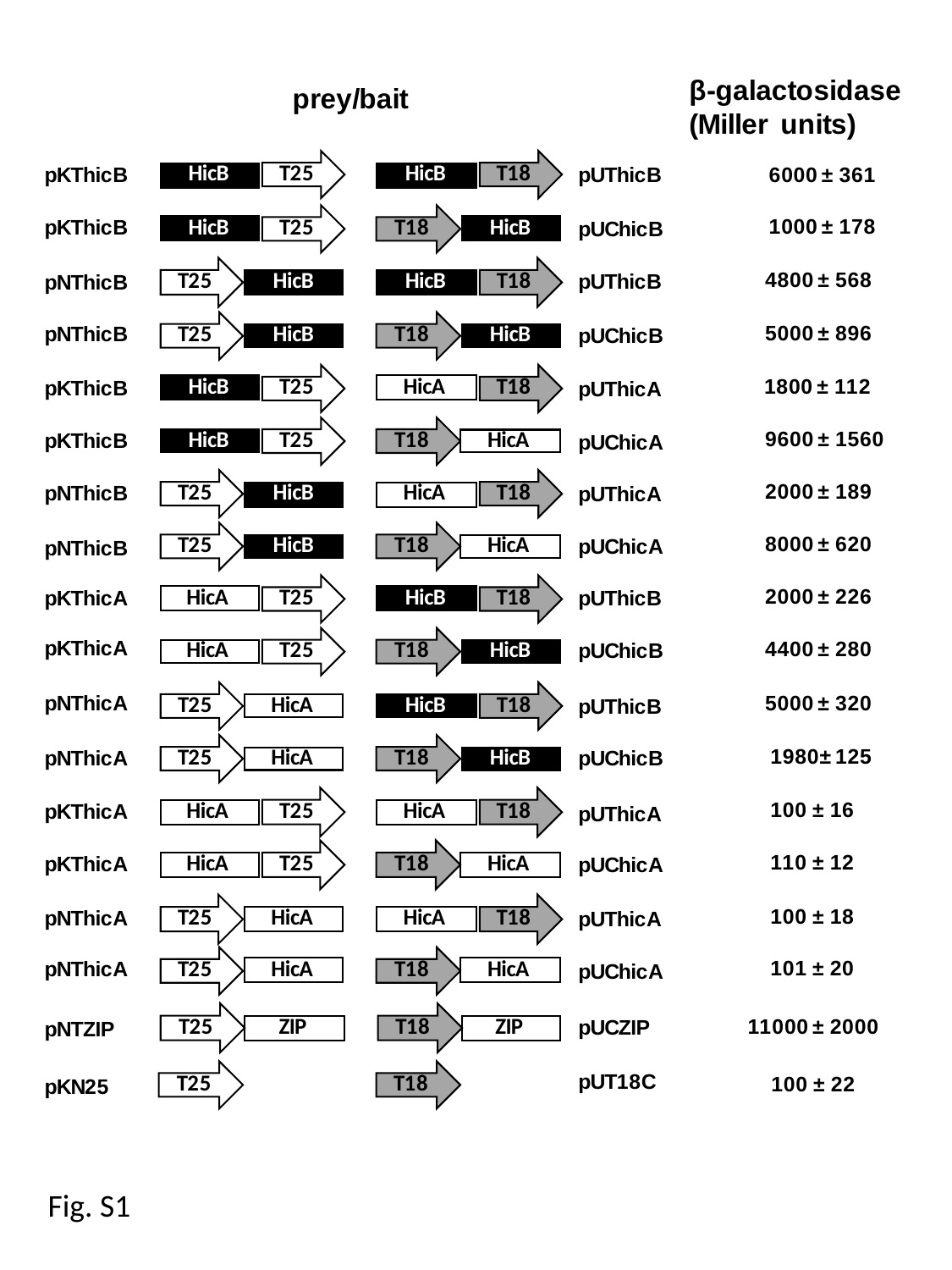

Fig. S1

Supplement: Supplementary file 1 — Figure S1. HicA/HicB interaction analysis using the BACTH system. For analysis of interactions between HicA and HicB, either one of the plasmids pKThicA, pNThicA, pKThicB, or pNThicB were transformed into the E. coli strain BTH101 reporter strains (a cya-deficient strain), followed by secondary transformation of any of the following: pUThicA, pUChicA, pUThiB, or pUThicB. Positive and negative controls were performed using pKT25-ZIP/pUT18-ZIP and pKNT25/pUT18C sets respectively. Positive interactions allow the reconstitution of adenylate cyclase activity and thus the expression of lacZ gene in the cya strain BTH101. β–galactosidase activity (Miller units) is the mean of three independent experiments. (PPTX 95 kb) [file 12866_2018_1382_MOESM1_ESM.pptx]

## Slide 1
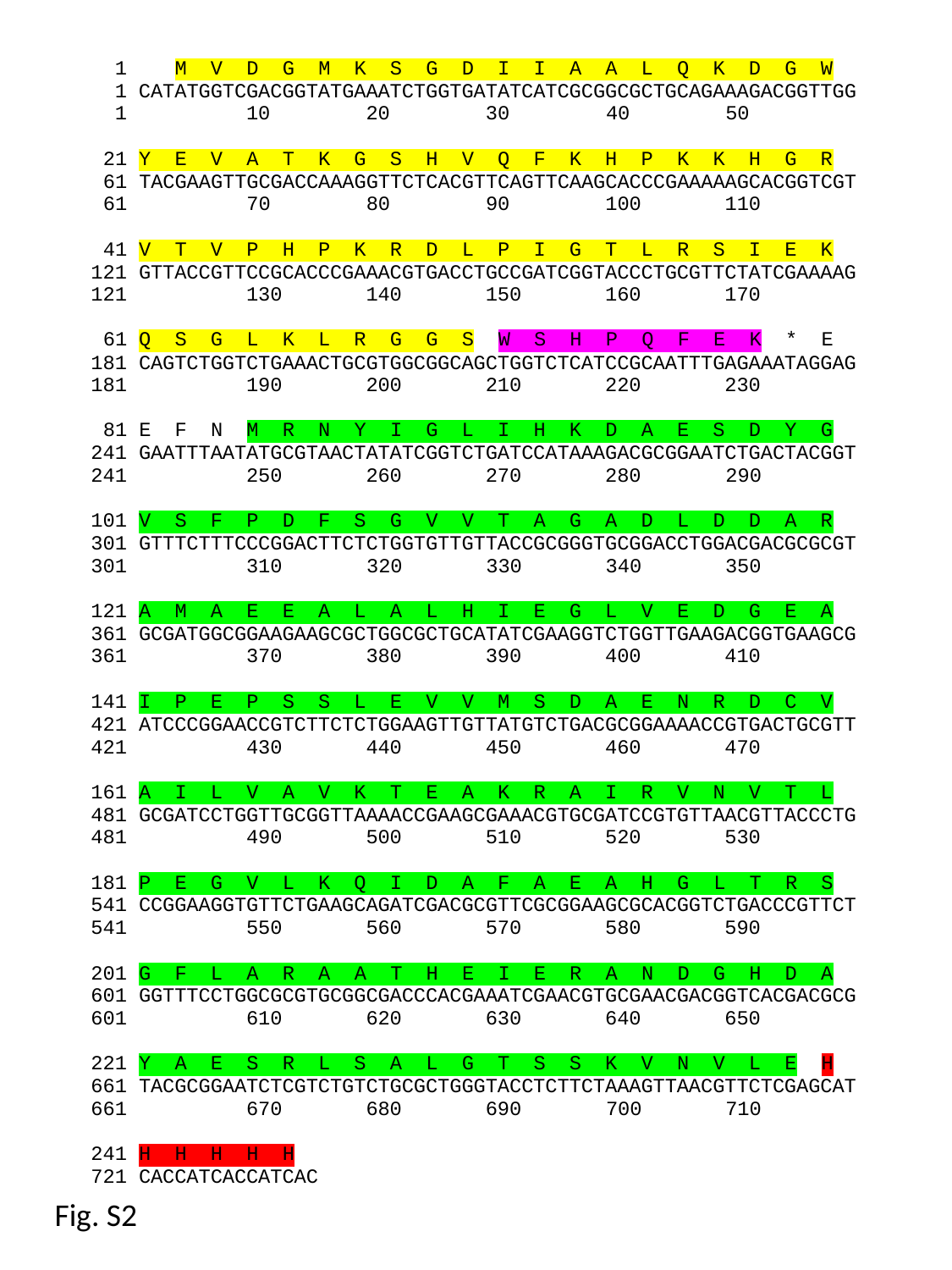

Fig. S2

Supplement: Supplementary file 2 — Figure S2. Nucleotide sequence of hicA-hicB synthetic operon introduced into pEt22b(+). HicA residues are highlighted in yellow, strep tag in pink, HicB in green and his tag in red. (PPTX 99 kb) [file 12866_2018_1382_MOESM2_ESM.pptx]

## Slide 1
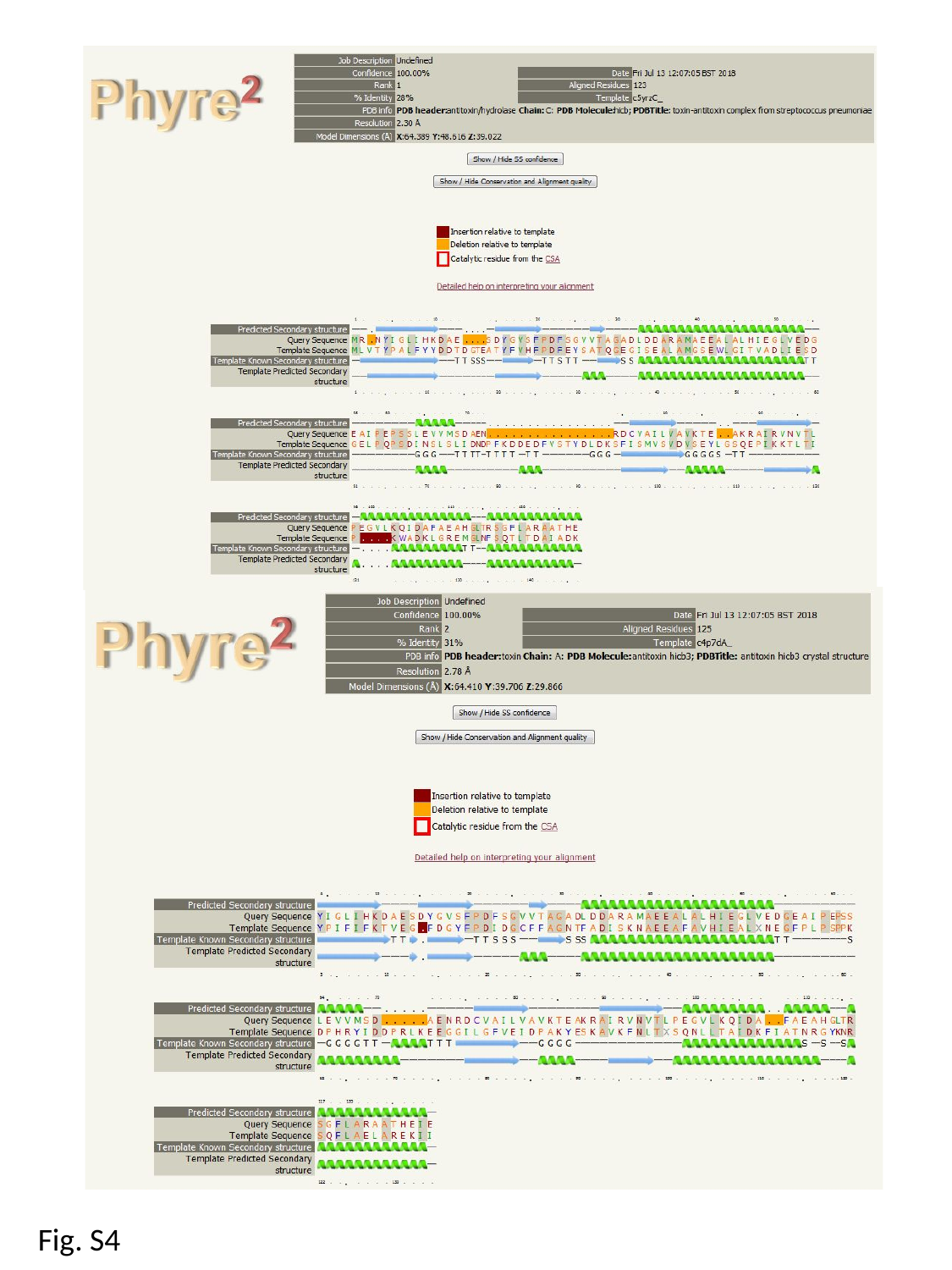

Fig. S4

Supplement: Supplementary file 4 — Figure S4. Alignments of S. meliloti HicB with HicB of S. pneumoniae (A)and HicB3 of Y. pestis (B) using Phyre [44]. (PPTX 598 kb) [file 12866_2018_1382_MOESM4_ESM.pptx]

## Slide 1
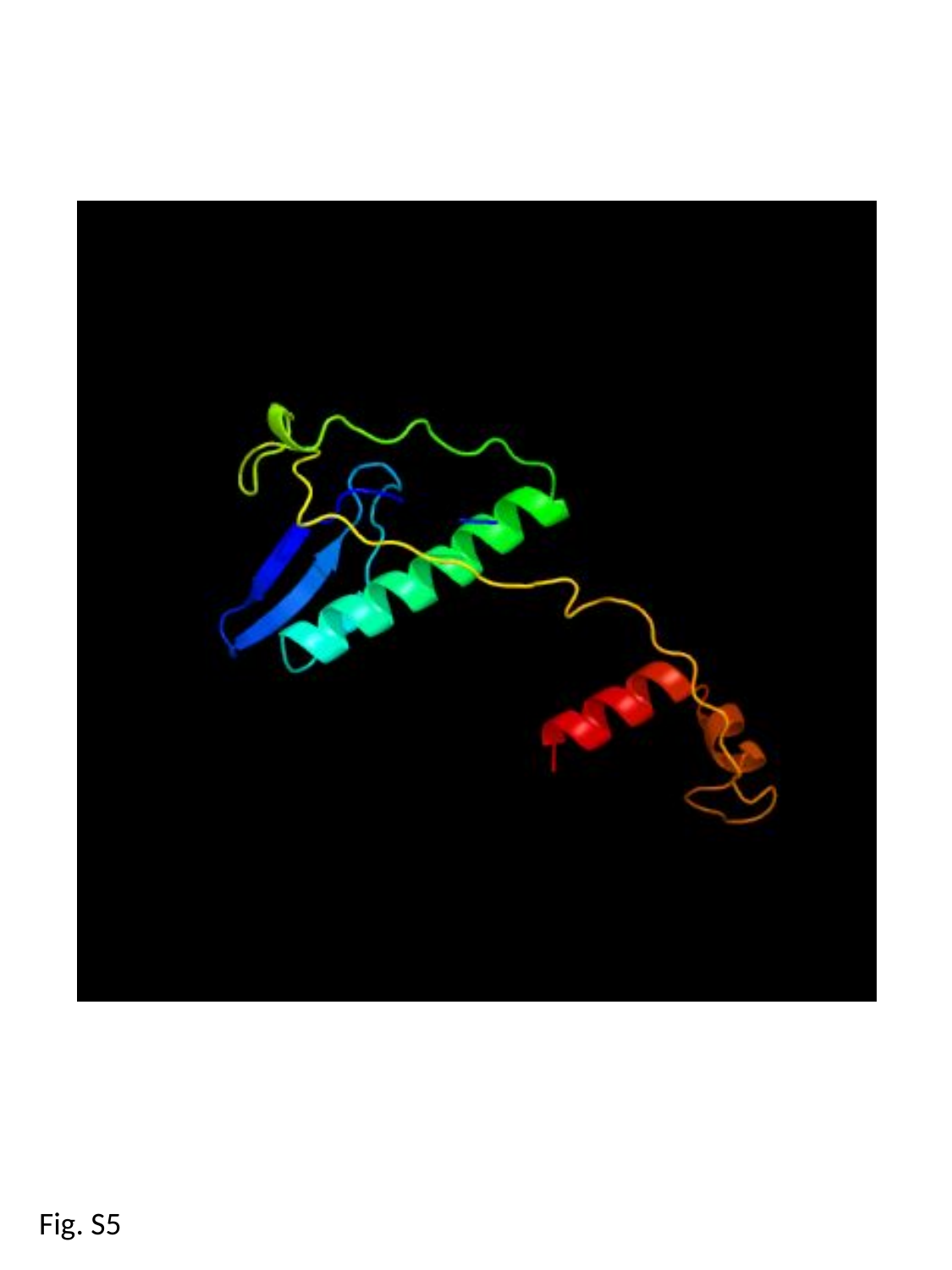

Fig. S5

Supplement: Supplementary file 5 — Figure S5. S. meliloti HicB structure predicted by Phyre using HicB of Y. pestis and S. pneumoniae as template. (PPTX 80 kb) [file 12866_2018_1382_MOESM5_ESM.pptx]

## Slide 1
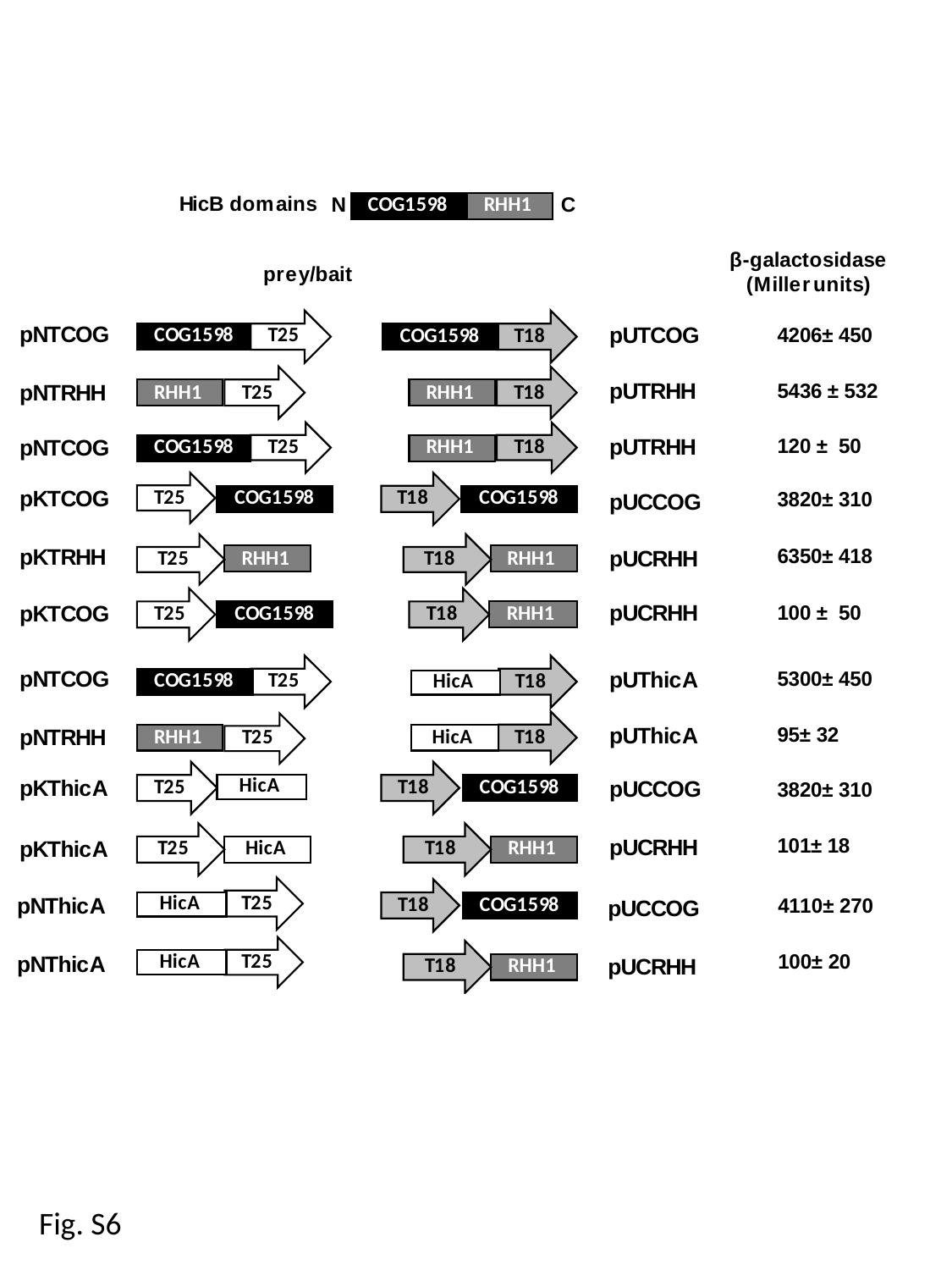

Fig. S6

Supplement: Supplementary file 6 — Figure S6. HicB domains interactions. For analysis of auto-interactions between HicB domains and their interaction with HicA, either one of the plasmids pKTCOG, pNTCOG, pKTRHH, pNTRHH, pKThicA or pNThicA were transformed into the E. coli strain BTH101 reporter strains (a cya-deficient strain), followed by secondary transformation of any of the following: pUTCOG, pUCCOG, pUTRHH, pUTCRHH or pUThicA. Positive and negative controls were performed using pKT25-ZIP/pUT18-ZIP and pKNT25/pUT18 sets respectively (not shown). Positive interactions allow the reconstitution of adenylate cyclase activity and thus the expression of lacZ gene in the cya strain BTH101. β–galactosidase activity (Miller units) is the mean of three independent experiments. (PPTX 81 kb) [file 12866_2018_1382_MOESM6_ESM.pptx]
