# Supplementary material for: Characterization of HicAB toxin-antitoxin module of Sinorhizobium meliloti
Source: BMC Microbiol. 2019 Jan 10;19:10. doi: 10.1186/s12866-018-1382-6 (PMC6327479; doi:10.1186/s12866-018-1382-6)
Supplement: Supplementary file 3 — Figure S3. Alignments of HicA and HicB proteins with HicA and HicB homologues of E. coli, Y. pestis and S. pneumoniae using Clustal Omega [43]. Symbols indicate a conserved residue (*), conservative mutation (:) and a semi-conservative mutation (.). The H23 residue critical for HicA activity [25] is shown in red. (PPTX 55 kb) [file 12866_2018_1382_MOESM3_ESM.pptx]

## Slide 1
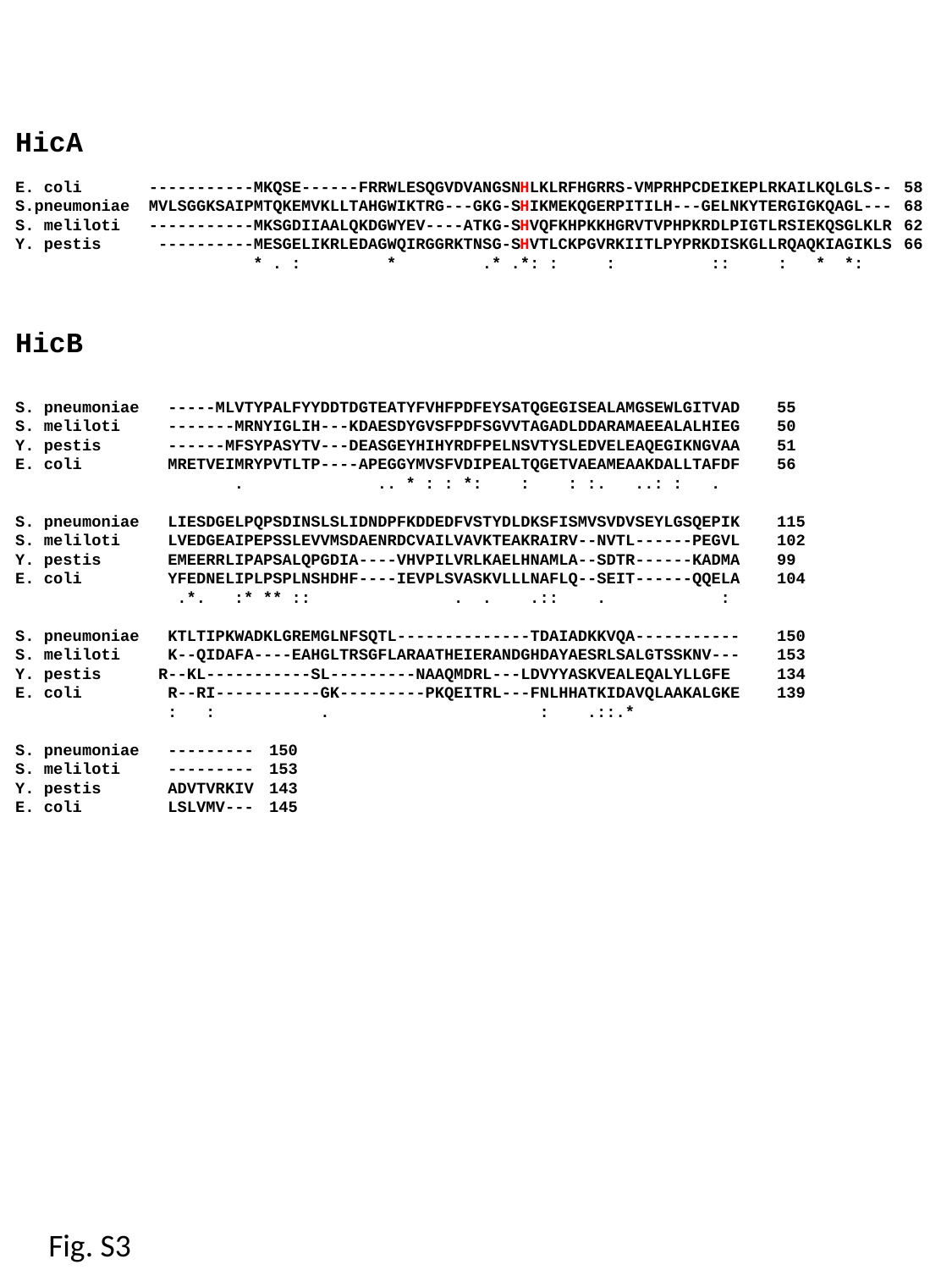

HicA
E. coli -----------MKQSE------FRRWLESQGVDVANGSNHLKLRFHGRRS-VMPRHPCDEIKEPLRKAILKQLGLS--	58
S.pneumoniae MVLSGGKSAIPMTQKEMVKLLTAHGWIKTRG---GKG-SHIKMEKQGERPITILH---GELNKYTERGIGKQAGL---	68
S. meliloti -----------MKSGDIIAALQKDGWYEV----ATKG-SHVQFKHPKKHGRVTVPHPKRDLPIGTLRSIEKQSGLKLR	62
Y. pestis ----------MESGELIKRLEDAGWQIRGGRKTNSG-SHVTLCKPGVRKIITLPYPRKDISKGLLRQAQKIAGIKLS	66
 * . : * .* .*: : : :: : * *:
HicB
S. pneumoniae -----MLVTYPALFYYDDTDGTEATYFVHFPDFEYSATQGEGISEALAMGSEWLGITVAD	55
S. meliloti -------MRNYIGLIH---KDAESDYGVSFPDFSGVVTAGADLDDARAMAEEALALHIEG	50
Y. pestis ------MFSYPASYTV---DEASGEYHIHYRDFPELNSVTYSLEDVELEAQEGIKNGVAA	51
E. coli MRETVEIMRYPVTLTP----APEGGYMVSFVDIPEALTQGETVAEAMEAAKDALLTAFDF	56
 . .. * : : *: : : :. ..: : .
S. pneumoniae LIESDGELPQPSDINSLSLIDNDPFKDDEDFVSTYDLDKSFISMVSVDVSEYLGSQEPIK	115
S. meliloti LVEDGEAIPEPSSLEVVMSDAENRDCVAILVAVKTEAKRAIRV--NVTL------PEGVL	102
Y. pestis EMEERRLIPAPSALQPGDIA----VHVPILVRLKAELHNAMLA--SDTR------KADMA	99
E. coli YFEDNELIPLPSPLNSHDHF----IEVPLSVASKVLLLNAFLQ--SEIT------QQELA	104
 .*. :* ** :: . . .:: . :
S. pneumoniae KTLTIPKWADKLGREMGLNFSQTL--------------TDAIADKKVQA-----------	150
S. meliloti K--QIDAFA----EAHGLTRSGFLARAATHEIERANDGHDAYAESRLSALGTSSKNV---	153
Y. pestis R--KL-----------SL---------NAAQMDRL---LDVYYASKVEALEQALYLLGFE	134
E. coli R--RI-----------GK---------PKQEITRL---FNLHHATKIDAVQLAAKALGKE	139
 : : . : .::.*
S. pneumoniae ---------	150
S. meliloti ---------	153
Y. pestis ADVTVRKIV	143
E. coli LSLVMV---	145
Fig. S3
